# Capturing variation in metagenomic assembly graphs with MetaCortex

**DOI:** 10.1093/bioinformatics/btad020

**Published:** 2023-01-12

**Authors:** Samuel Martin, Martin Ayling, Livia Patrono, Mario Caccamo, Pablo Murcia, Richard M Leggett

**Affiliations:** Earlham Institute, Norwich NR4 7UZ, UK; Earlham Institute, Norwich NR4 7UZ, UK; Robert Koch Institute, 13353 Berlin, Germany; NIAB EMR, Kent ME19 6BJ, UK; MRC-University of Glasgow Centre for Virus Research, Glasgow G61 1QH, UK; Earlham Institute, Norwich NR4 7UZ, UK

## Abstract

**Motivation:**

The assembly of contiguous sequence from metagenomic samples presents a particular challenge, due to the presence of multiple species, often closely related, at varying levels of abundance. Capturing diversity within species, for example, viral haplotypes, or bacterial strain-level diversity, is even more challenging.

**Results:**

We present MetaCortex, a metagenome assembler that captures intra-species diversity by searching for signatures of local variation along assembled sequences in the underlying assembly graph and outputting these sequences in sequence graph format. We show that MetaCortex produces accurate assemblies with higher genome coverage and contiguity than other popular metagenomic assemblers on mock viral communities with high levels of strain-level diversity and on simulated communities containing simulated strains.

**Availability and implementation:**

Source code is freely available to download from https://github.com/SR-Martin/metacortex, is implemented in C and supported on MacOS and Linux. The version used for the results presented in this article is available at doi.org/10.5281/zenodo.7273627.

**Supplementary information:**

Supplementary data are available at *Bioinformatics* online.

## 1 Introduction

The well-documented increase in yield and reduction in the cost of DNA sequencing technologies has led to a rapid increase in the use of shotgun approaches for studying metagenomic samples (Mitchell *et al.*, 2018). A first analysis step is often the taxonomic classification of reads by comparison with reference databases. However, deeper analysis is enabled by assembling sequence data to form a longer contiguous sequence (contigs). Such assembly may facilitate improved classification, clustering of sequences (particularly where reference genomes are unavailable) or analysis at the scale of genes.

There are two fundamental approaches for the assembly of sequencing data: overlap-layout-consensus (OLC) assembly and de Bruijn graph assembly. We recommend the reader consult (Ayling, 2020) for a more thorough overview. In OLC assembly, each read is compared to every other read and reads that overlap well are merged together to form contigs. The de Bruijn graph technique utilizes directed graphs to represent the k-mers (short sequences of length *k*) present in a set of reads, and this representation of the read set turns the assembly problem into a graph traversal problem. Traversing a graph has a lower order of time complexity than OLC and so the computational time to perform an assembly can be significantly reduced. Furthermore, the amount of memory required to build the graph is proportional to the total k-mers present in the sample, rather than the total number of reads. This is particularly important as new generations of sequencing technologies are producing ever greater amounts of data.

Metagenomic assembly presents several challenges beyond those of *de novo* genomic assembly. The main additional difficulties are unknown diversity and unknown species abundance (Ayling, 2020). Given two sufficiently distinct genomes, one can find a (possibly very large) k-mer size such that the genomes do not share k-mers. Thus, it can be inferred that for large enough k, subgraphs representing distinct species are mostly disconnected and existing assembly techniques from *de novo* genome assembly may be used on each disconnected subgraph. However, in practice, such a *k* is likely to be far larger than the read size, and so impractical for de Bruijn graph construction. Furthermore, it is often not the case that a metagenomic sample consists solely of diverse species, and separating closely related species, or many strains of a single species, is a difficult task, particularly at low abundance.

The problem of capturing diversity below the species level in an assembly presents an even bigger challenge. The genomes of different strains from a single species can differ by single nucleotide polymorphisms (SNPs), large-scale structural variation and anything in between. In many ways, the challenges faced here are similar to those faced when separating haplotypes in *de novo* assembly, where much of the same sequence is shared between haplotypes. In this case, one can use the ploidy of the organism and the k-mer coverages to guide the assembly. However, in the metagenomic case, we do not necessarily know what the expected coverage of each strain is, and in low abundance cases, it will be difficult to distinguish a SNP from a sequencing error (Quince, 2021). The challenge is at its most stark when studying viral metagenomes. Due to their short replication times, large population sizes and lack of proofreading mechanisms [coronaviruses are a notable exception (Denison, 2011)], viruses can evolve extremely rapidly. As such, viruses are often referred to as a quasispecies consisting of a set of related strains; and the genome sequence of a strain is sometimes referred to as a haplotype (Gregori, 2016).

Due to the throughput of current next-generation sequencing technologies, it is possible to have a metagenomic dataset consisting of several terabytes of reads [e.g. the NovaSeq 6000 is capable of producing up to 6000 Gb of sequencing data in a single run (https://www.illumina.com/systems/sequencing-platforms.html, accessed September 2022)]. Many assemblers are incapable of assembling such large datasets within realistic time and memory constraints. One strategy to make the problem tractable is to subsample the read set to obtain a much smaller one which can then be assembled. This process will usually not affect the assemblies of high-abundance species in the sample [in fact, it can improve them (Hug, 2016)], but there is evidence that the assemblies of the low-abundance species will be incomplete and of poorer quality (Cattonaro, 2018).

Many current short-read metagenomic assemblers utilize the de Bruijn graph paradigm. Popular examples include Ray Meta (Boisvert, 2012), MEGAHIT (Li, 2015), MetaVelvet (Namiki, 2012) and metaSPAdes (Nurk, 2017). A key part of the implementation of all these tools is to collapse an assembly graph into linear sequences (usually in the form of a FASTA file). This facilitates easy downstream analysis, but the act of converting the assembly graph into a linear sequence has the effect of removing the understanding of sequence diversity that is implicit in the graph (Brown, 2020). Several assemblers have been created specifically for the assembly of viral quasispecies, such as SAVAGE (Baaijens, 2017) and VICUNA (Yang, 2012). These tools are able to assemble the haplotypes present in an isolated sample but may be less effective at assembling quasi-species from metagenomic samples. Within the genome assembly world, there is a growing awareness of the importance of capturing the genome graph in the output from assembly tools. This has resulted in the development of the FASTG format (http://fastg.sourceforge.net, accessed 25 May 2021) and, more recently and with wider adoption, the graphical fragment assembly (GFA) format for assembly graph files (https://github.com/GFA-spec/GFA-spec, accessed 25 May 2021). These have been implemented in a number of tools including recent versions of ABySS (Jackman, 2017), SPAdes (Bankevich, 2012), metaSPAdes and SDG (Yanes, 2019). Other tools have addressed the problem of analysing the quantity of sequencing data that is currently available, particularly in metagenomics. For example, MetaGraph can construct a de Bruijn graph from petabases of sequencing data for sequence querying and assembly (Karasikov, 2020). Within the metagenomics world, several recent tools have focused on identifying and assembling strain-level variation from assembly graphs. STRONG uses multiple metagenome samples from a time series to identify strains *de novo* from an assembly graph, and performs coassembly and genome binning (Quince, 2021). The tool spacegraphcats can perform a local search of an assembly graph to identify variation that is not present in reference sequences (Brown, 2020), and the tool KOMB uses assembly graphs to identify copy number variants and structural variants in a metagenomic read set (Balaji, 2022).

Here, we introduce MetaCortex, a de Bruijn graph metagenomic assembler that is built upon data structures and graph-traversal algorithms developed for the Cortex assembler (Iqbal, 2012). As well as performing metagenomic assembly with standard FASTA output, MetaCortex generates sequence graph files that preserve intra-species variation (e.g. viral haplotypes) and implements a new graph traversal algorithm to output variant contig sequences. Whilst MetaCortex can be used to assemble any metagenomic dataset, we have developed features to specifically target metagenomic datasets with high levels of strain diversity (e.g. viral communities) and to represent this diversity in the resulting assembly. MetaCortex captures variation by looking for signatures of polymorphisms in the de Bruijn graph constructed from the reads and represents this in sequence graph format (both FASTG and GFA v2) and the usual FASTA format. The sequence graph provides information on local variation, such as SNPs and indels, along each contig identified by MetaCortex. By using the efficient data structures from Cortex, MetaCortex is capable of utilizing all k-mers from large metagenomic datasets and able to perform assemblies from these datasets on a single CPU. One of the novel features of Cortex was to introduce coloured de Bruijn graphs. This is not yet utilized by MetaCortex, but the code has been written to allow an easy implementation in future versions.

We show that MetaCortex is able to produce highly contiguous assemblies capturing almost all genome level diversity and with a low level of misassemblies. By outputting sequence graph files, we were able to capture strain-level diversity that is not present in the contigs and use this to manually assemble contigs that were specific to individual strains in a sample.

## 2 Materials and methods

To test MetaCortex, we assembled real Illumina read sets from two mock communities of 12 viruses, at varying levels of abundance; a real human gut sample which was subsequently *in silico* mixed with real reads from a lab mix of five strains of HIV in equal abundance; and two simulated read sets from communities with high levels of strain variation. In each case, we assembled the dataset using the Subtractive Walk (SW) algorithm with delta value equal to 0.8 (see Section 3). The minimum coverage parameter was set to 10, except for the lower coverage simulated datasets, where it was set to 5. The parameters for the HIV dataset were adjusted to suit this dataset.

To compare MetaCortex’s performance with that of existing *de novo* metagenome assemblers, we also assembled the same datasets using MEGAHIT (v1.1.1), Ray Meta (v2.3.1), MetaVelvet (v1.2.02), metaSPAdes (v3.14) and IDBA-UD (v1.1.2) (Peng, 2012). For each assembler, we assembled each dataset using a range of parameters where these were available. For MetaCortex and Ray Meta, we used k-mer values equal to 31, 63, 95 and 127. For MetaVelvet, we used k-mer values equal to 31, 63 and 95. Since MEGAHIT uses several k-mer values when constructing an assembly, we varied the –min-count parameter (with values default, 5, 10 and 20) for each dataset. The assemblies by metaSPAdes were created using the default options. For the real sequence data, we used trim-galore v0.5.0 (https://github.com/FelixKrueger/TrimGalore, accessed November 2020) [a wrapper script around Cutadapt (Martin, 2011)], to trim low-quality bases and adapter sequence from the reads, with the flags –paired and –retain_unpaired where appropriate. Since this can break the pairing of paired-end reads and result in single-ended reads, we assembled these sets using both ‘paired + unpaired single’ and ‘single only’ modes, for the assemblers where this was possible. The assemblies by IDBA-UD either failed, or were still running after 62 days, so the results for this assembler are not provided.

Statistics on the assemblies were obtained using MetaQUAST (Mikheenko, 2016), which by default considers only those contigs greater than 500 bp in length. A position on a contig is considered a misassembly by MetaQUAST if either the left flanking sequence aligns over 1 kb away from the right flanking sequence on a reference genome; flanking sequences overlap on more than 1 kb; or flanking sequences align to different strands, chromosomes or genomes (i.e. chimeric assemblies). We set the flag ‘ambiguity-usage’ equal to one, so that only the best alignment from each contig is used when calculating certain statistics. Here, for each dataset, we present a single assembly from each assembler, that we judged to be the best (based on genome coverage and error rates). Full results for all assemblies are available in the Supplementary Materials, along with the commands used.

### 2.1 Assembly of sequenced mock viral communities

For our first benchmark, we assessed how well MetaCortex performs metagenomic assembly on simple mock communities compared to other current assemblers. Two mock communities of 12 viruses, each containing 2 ssDNA viruses and 10 dsDNA viruses, were assembled from real Illumina read data made available for benchmarking purposes (Roux, 2016).

For both mocks, IDBA-UD was still running after 60 days, with 8 CPUs assigned. The assemblies by MetaVelvet were either killed by the Linux Out of Memory (OOM) killer after running out of memory, with 3TB of RAM allocated, or they recovered an insignificant total genome fraction, and so are not reported here. For MockB, metaSPAdes failed after running out of memory, with 3TB of RAM allocated. This is beyond the memory limits for many researchers, and the usual strategy at this point is to either use an assembler with lower resource requirements [such as MEGAHIT, as in e.g. Kim (2021)] or assemble a much smaller subsample of reads instead. However, recent studies suggest that subsampling can drastically reduce the length and proportion of conserved genes in the subsequent assembly when compared to the assembly of the full dataset (Cattonaro, 2018).

**Fig. 1.**
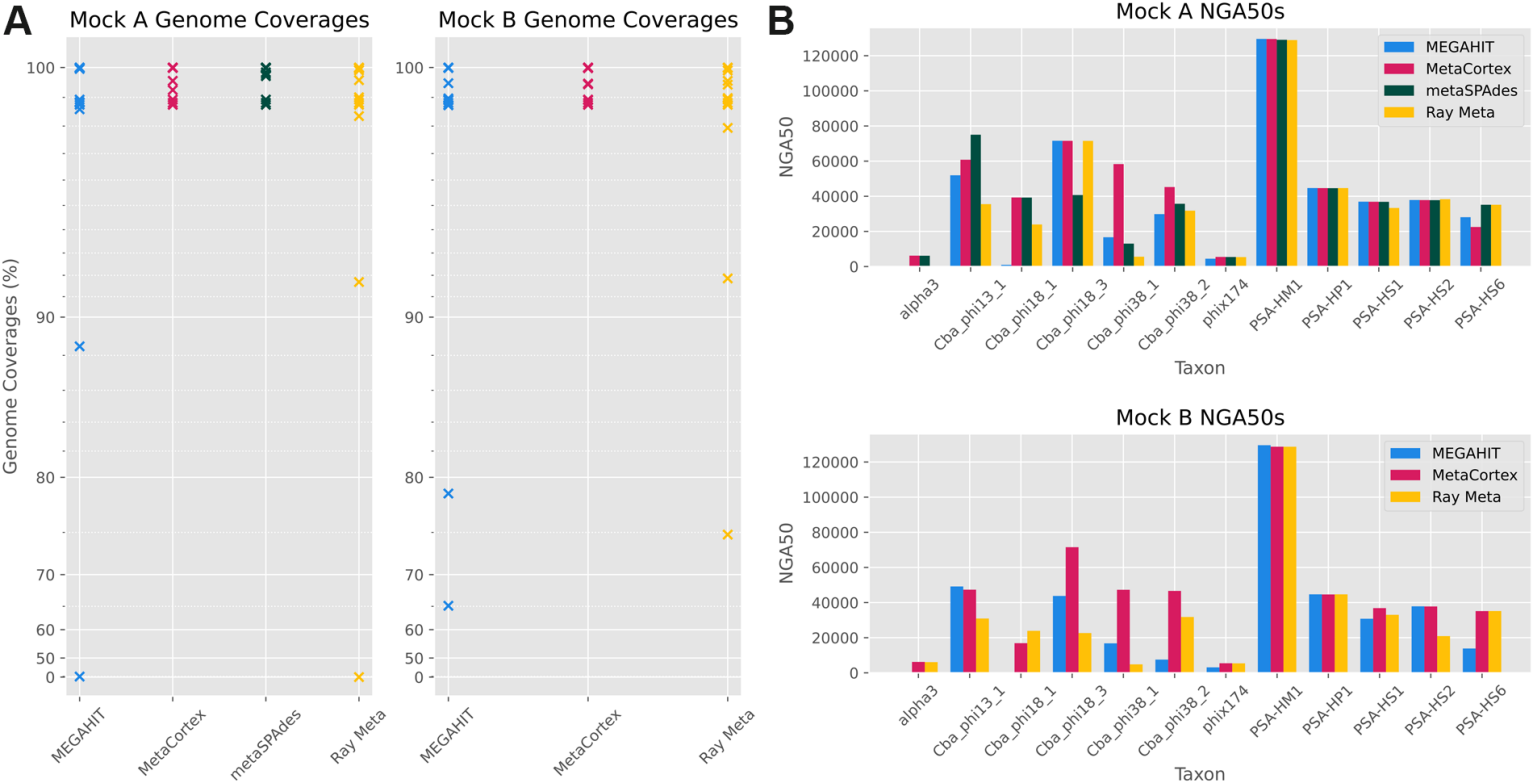
(**A**) Genome coverages for Mock A and Mock B for assemblies presented in Tables 1 and 2. Each cross indicates the assembled percent of a genome in the mock. A cross at 0% indicates that a known genome in the sample was not present in the assembly. (**B**) NGA50 by viral species for assemblies presented in Tables 1 and 2

The first community, Mock A, was composed of the dsDNA viruses each (theoretically) at 9.82% abundance and the ssDNA viruses at 0.92%. The read set consisted of 2 × 250 bp paired-end reads, sequenced on the Illumina MiSeq platform, with a total read count of 96 m, including reads from host DNA. Using MetaCortex (*k* = 95), we were able to assemble 99.89% of the viral genomes with no misassemblies. Mismatch and indel rates were very low, at 4.43 per 100 kb and 3.44 per 100 kb, respectively. Individual genome coverages ranged from 100% to 99.56%. Eight virus genomes were each assembled in a single contig, while the assembly of all other genomes ranged from 8 to 40 contigs. Figure 1B shows that the assembly by MetaCortex is the most contiguous.

Table 1 shows the assembly statistics as produced by MEGAHIT (paired, min count = 20), MetaCortex (*k* = 95), metaSPAdes (default) and Ray Meta (paired, *k *= 31). Only the assembly by metaSPAdes recovers a higher genome fraction than the assembly by MetaCortex, with an extra 0.012%. However, metaSPAdes recovered only four species at 100% (compared with six for MetaCortex) and seven in a single contig (compared with eight for MetaCortex). The assembly by MetaCortex was the most contiguous (Fig. 1B) and has error rates almost identical to metaSPAdes (which had the lowest), with only a slightly higher indel rate. The assembly by MEGAHIT recovered a similar genome fraction across most species in the mock (Fig. 1A), but the assembly was less contiguous (Fig. 1B) and had the highest mismatch rate and the most misassemblies. Notably, the assembly by Ray Meta failed to assemble any of the genome of the ssDNA phage alpha3.

**Table 1.** Summary statistics for assemblies of Mock A

	Genome fraction (%)	Genomes covered > 50%	Contigs	Misassemblies	Mismatch rate	Indel rate
MEGAHIT	98.233	11/12	1902	3	75.7	3.33
MetaCortex	99.886	12/12	339	0	4.43	3.44
metaSPAdes	99.898	12/12	2259	0	4.43	3.12
Ray Meta	97.652	11/12	1737	0	9.06	3.02

*Note*: The contigs field describes the number of contigs in the assembly of length greater than or equal to 500 bp. Misassemblies are defined in the text above and include chimeric assemblies. Mismatch and indel rates are the number of occurrences per 100 kb. The NGA50 for each genome in the sample can be found in Figure 1B.

The total aligned length of the assembly by MetaCortex was 1.09 Mb, out of a total assembly length of 5.7 Mb. We used BLAST to query the longest unaligned contigs (greater than 100 kb) against the nt database. This revealed that each had alignments to one of Pseudoalteromonas or *Cellulophaga baltica*. These were host species that the mock community was grown on, so this represents legitimate assembly of DNA present in the sample. Including these species in the reference list for MetaQUAST, we had a total aligned length of 4.96 Mb, of which 3.87 Mb aligned to *C.baltica* over 143 contigs (covering 82.03% of the *C.baltica* genome).

The second community, Mock B, consisted of the dsDNA viruses at 3.51% abundance, and the two ssDNA viruses each at 32.47% abundance. The read set consisted of 98m 2 × 250 bp paired-end reads. Table 2 shows the assembly statistics as produced by MEGAHIT (single, min count = 20), MetaCortex (*k* = 63) and Ray Meta (single, *k* = 31). Using MetaCortex we assembled 99.71% of the community, with a single misassembly. The percent of individual genomes recovered ranged from 100% (for five genomes) to 98.744%. Seven virus genomes were assembled in a single contig, and the assembly of all other genomes varied from between 6 and 25 contigs. As was the case in Mock A, ∼3.80 Mb of unaligned contigs were found to align to *C.baltica*, covering 81.804% of its genome.

**Table 2.** Summary statistics for assemblies of Mock B

	Genome fraction (%)	Genomes covered > 50%	Contigs	Misassemblies	Mismatch rate	Indel rate
MEGAHIT	94.15	11/12	1762	2	15.66	3.65
MetaCortex	99.711	12/12	735	1	7.23	3.45
Ray Meta	95.582	12/12	2240	0	4.63	2.91

MetaCortex recovered the largest genome fraction, with a similar fraction recovered to Mock A. The assembly by MetaCortex was also the most contiguous (Fig. 1B), although this time it contained a single misassembly (one more than Ray Meta) and had error rates between those in the assemblies by Ray Meta and MEGAHIT (Table 2).

### 2.2 Assembly of sequenced HIV lab mix

In order to evaluate MetaCortex’s ability to capture variants in a real metagenomic sample, we created a read set containing five well-studied strains of HIV-1 (89.6, HXB2, JR-CSF, NL4-3 and YU2) in equal abundance that was bioinformatically mixed with reads from a human preterm baby gut sample. Reads and reference sequences for each HIV strain were made available in Di Giallonardo (2014) (SRA run SRR961514). Each strain was between 93% and 97% identical to the other strains, and the read set consisted of 308 Mb of Illumina 2 × 250 bp reads. Reads from the human gut sample were taken from (Leggett, 2020) (ENA run accession ERR2099157), consisting of 10.5 Gb of Illumina 2 × 250 bp reads. After mixing, reads from the HIV mix consisted of about 2.8%, representing about 0.56% per strain.

To increase the sensitivity of MetaCortex’s ability to capture strain-level variation, we set the SW delta parameter to 0.4 and the min coverage parameter to 25. With these values, MetaCortex (SW, *k* = 127) assembled 82.563% of the five HIV genomes across 31 contigs with no misassemblies.

**Fig. 2.**
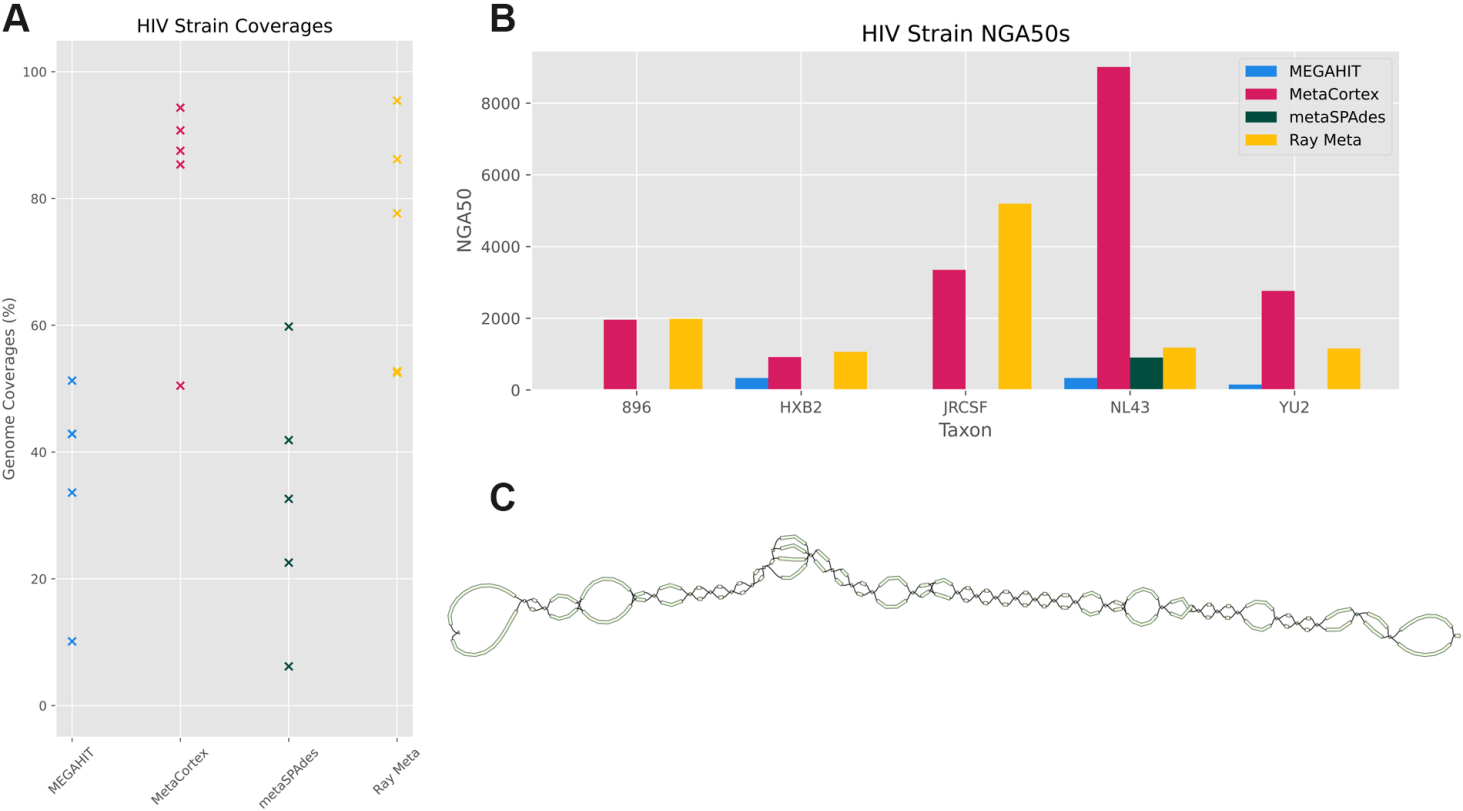
Assemblies of HIV-5-strain dataset. (**A**) Coverage for each viral species for each assembler. (**B**) NGA50s for each strain per assembler. (**C**) Visualization of GFA sequence graph output from MetaCortex. The sequence graph shows a single contig assembled by MetaCortex, with local variation shown by branching sequences

Table 3 shows the best assemblies as produced by MEGAHIT (paired, min count = 5), MetaCortex (*k* = 127), metaSPAdes (default), MetaVelvet (*k* = 95) and Ray Meta (paired, *k* = 127). Figure 2A shows the individual coverages for each of the strains in these assemblies, and Fig. 2B shows the NGA50s. The assembly by MetaCortex produced the assembly with the highest genome fraction and fewest misassemblies. Only Ray Meta produced an assembly with a significantly lower mismatch rate, but this contained more misassemblies and a smaller genome fraction. Another assembly by Ray Meta (see Supplementary Materials) contained a smaller genome fraction (70.089%) with no misassemblies but higher mismatch and indel rates (76.67 and 11.8, respectively).

**Table 3.** Summary statistics for assemblies of 5-strain HIV mix

	Genome fraction (%)	Contigs	HIV-aligned contigs	Misassemblies	mismatch rate	Indel rate
MEGAHIT	36.153	5075	43	1	2744.27	68.61
MetaCortex	82.563	4635	31	0	508.2	10.1
metaSPAdes	36.153	3409	10	0	1424.3	0
MetaVelvet	2.011	3661	3	0	1747.17	0
Ray Meta	72.597	1243	43	1	74.03	8.54

#### 2.2.1 GFA output captures strain-level variation and facilitates visualization

**Fig. 3.**
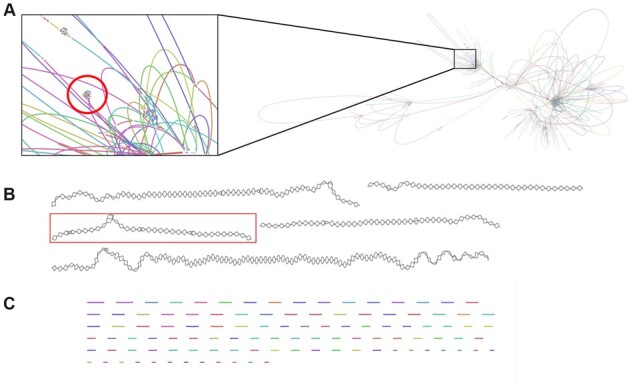
(**A**) Portion of assembly graph produced by metaSPAdes. Highlighted area shows sequences that mapped to the HIV reference genomes. (**B**) Portion of sequence graph produced by MetaCortex. Highlighted area represents the single contig that mapped to the HIV reference genomes. (**C**) FASTA output from Ray Meta represented as a sequence graph

As an alternative way to capture strain-level diversity, we assembled the same dataset as above using MetaCortex's MC algorithm, with the sequence graph output enabled and the minimum coverage parameter set to 25. This creates a sequence graph showing local variation along each contig in the assembly (see Fig. 3B). This allows us to examine local variation along the contigs identified by MetaCortex without having to disentangle the larger assembly graph. For comparison, Figure 3A highlights the contigs from the metaSPAdes assembly that map to the HIV genomes within the assembly graph output produced by metaSPAdes [and visualized using Bandage (Wick, 2015)]. These sequences are embedded within a much larger and more complex connected graph. On the other hand, Figure 3C represents the FASTA output from the Ray Meta assembly in sequence graph form. Here, we have no information about local variation and connectedness without performing further analyses.

Next, we used BLAST (Altschul, 1990) to map each contig against a database consisting of the reference genomes for each HIV strain. We found one contig of length 9166 bp (about the length of the HIV genome) that mapped well to the reference genomes. We extracted this contig, and the corresponding sequence graph elements into new files. Figure 2C shows the sequence graph corresponding to this contig from the MetaCortex sequence graph output, as visualized by GfaViz (Gonnella, 2019).

To demonstrate that this sequence graph contains more information than the corresponding contig, we constructed one contig for each of the five strains present in the sample. First, we created a mapping of each sequence in the graph to a database of the reference genomes using BLAST. Then, we constructed contigs in the following way. For each strain, a walk is performed through the sequence graph, where at each branch, the branch whose sequence has the highest score for the strain is chosen. Scores are calculated as the mapping identity multiplied by the proportion of the alignment covering the query (in ambiguous cases, the branch corresponding to the original contig is chosen). Each walk corresponds to a contig, which forms the strain-level contig. A python script to parse the GFA file and BLAST mapping and construct the individual strain assemblies is available in the MetaCortex repository, under scripts/strain_assembly.py.

Next, we used dnadiff [part of MUMmer (Kurtz, 2004)] to compare each strain-specific contig and the original contig to the strain’s reference sequence. We found that the strain-specific contigs had alignments with a higher average identity to the corresponding reference strain and contained far fewer SNPs and indels (Table 4).

**Table 4.** Summary statistics comparing each strain-specific assembly to the base assembly

	NL4-3		JR-CSF		HXB2		89.6		YU2	
	Original	Strain specific	Original	Strain specific	Original	Strain specific	Original	Strain specific	Original	Strain specific
Total length	9166	9125	9166	9166	9166	9126	9166	9139	9166	9155
Aligned bases ref (%)	100	100	100	100	100	100	99.98	99.98	100	100
Aligned bases query (%)	100	100	100	100	100	100	99.98	99.98	100	100
Avg identity	96.04	97.17	97.39	99.19	95.81	96.70	93.82	96.41	94.81	95.69
Total SNPs	278	192	180	75	318	259	419	226	394	330
Total indels	83	58	11	1	81	55	72	9	56	33

### 2.3 Assembly of simulated viral and bacterial datasets

We tested MetaCortex’s performance on two simulated communities, one viral and one bacterial, both with a high amount of strain-level variation and highly variable compositions. For both communities, we used the software CAMISIM (Fritz, 2019) to simulate the community composition, strain-level variants and 15 Gb of Illumina 2 × 150 bp paired-end reads, with a HiSeq 2500 error profile. The simulated viral reads had variable but very high coverage, with coverage ranging from 44370× to 143673× for individual taxa across 10 genomes. Previous studies have suggested that the ability of assembly tools to deal with ultra-high coverage genomes is an important but often under-appreciated aspect of virome analysis, particularly when using library preparation methods that increase overall sequencing depth in order to improve recovery of low abundance genomes (Sutton, 2019). The simulated bacterial reads had variable coverage, with coverage ranging from 0.38× to 2557× for individual taxa.

Using the reference genomes (both real and simulated), we were able to compare the performance of MetaCortex, MEGAHIT, Ray Meta, metaSPAdes and MetaVelvet, on these datasets. Since the reads were simulated without adapter sequence, we assembled them without adapter trimming and used only the paired-end assembly mode for Ray Meta and MEGAHIT.

**Fig. 4.**
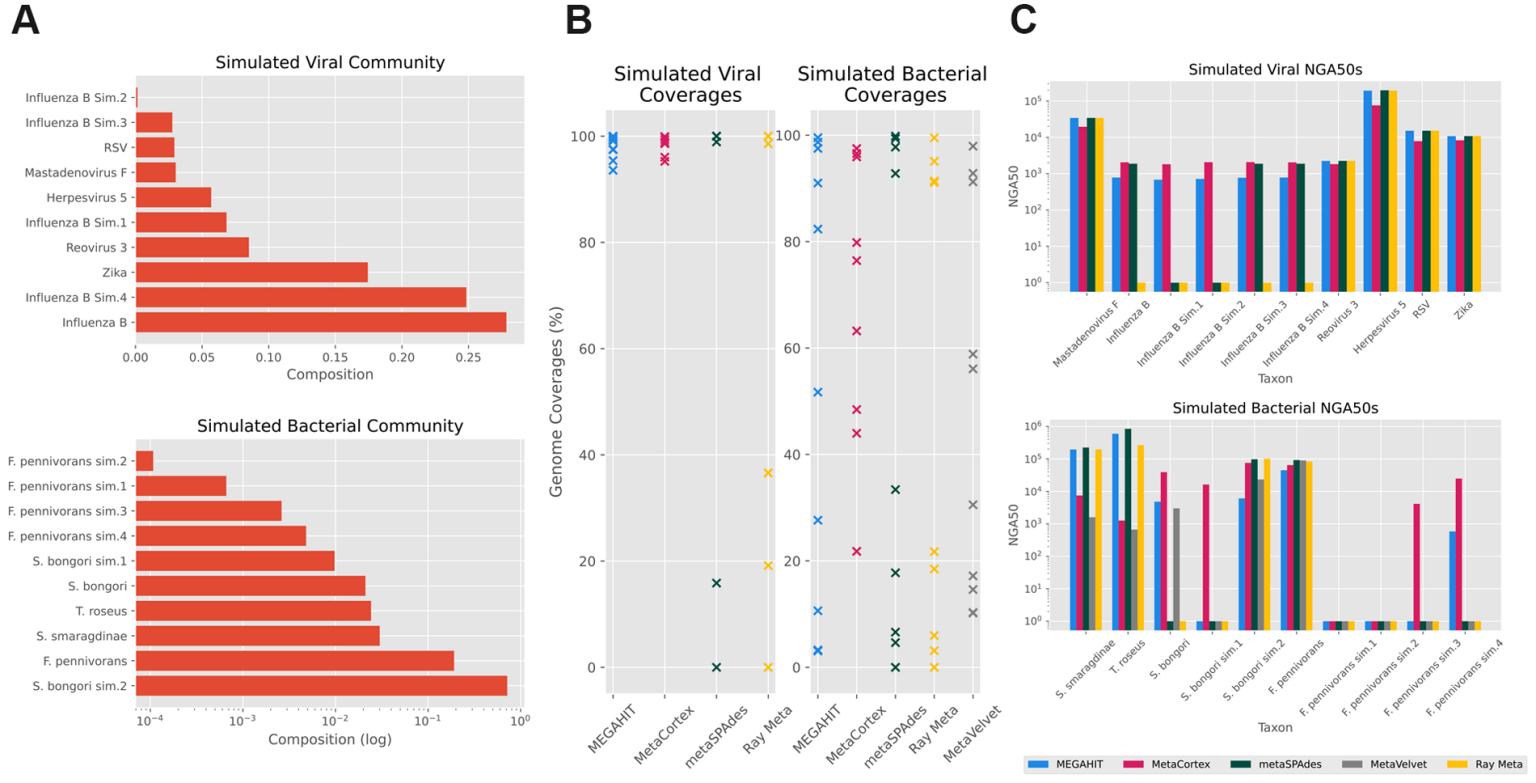
(**A**) Compositions of simulated communities. (**B**) Coverage per species for each assembler. (**C**) NGA50s of each species by assembler (log scale)

The viral community consisted of six species: Human mastadenovirus F, Human herpesvirus 5, Human respiratory syncytial virus, Influenza B virus, Reovirus 3 and Zika virus; and four simulated strains of Influenza B virus. Each simulated strain had between 99.93% and 99.95% of bases aligned to the genome it was simulated from, with an average alignment identity of between 97.09% and 99.63%. The composition of the community is shown in Figure 4A, and we simulated 150 bp long paired-end reads for a total of 14.7 Gb. The results in Table 5 show the best assemblies using parameters: MEGAHIT (paired, default parameters), MetaCortex (*k* = 63, min coverage = 5), metaSPAdes (default parameters) and Ray Meta (paired, *k* = 63).

**Table 5.** Summary statistics for assemblies of simulated viral community

	Genome fraction (%)	Genomes covered > 50%	Contigs	Misassemblies	Mismatch rate	Indel rate
MEGAHIT	98.709	10/10	1017	17	550.91	1.55
MetaCortex	98.777	10/10	2833	0	370.21	2.33
metaSPAdes	92.550	8/10	23	0	54.12	0.83
Ray Meta	77.241	3/10	14	3	10.59	0

For this dataset, MetaCortex recovered the highest overall genome fraction (98.78%), with individual genome fractions ranging from 95.27% to 99.91% and had no misassemblies. MEGAHIT recovered a similar genome fraction but had significantly more misassemblies and a higher mismatch rate. Both Ray Meta and metaSPAdes recovered smaller genome fractions (77–93%), although with lower error rates (Table 5). Individual genome coverage for each assembler is displayed in Figure 4B. MetaCortex achieved the highest NGA50 for the simulated strains (which were also the least abundant) but had lower NGA50 values for some of the other species (Fig. 4C). The assemblies by MetaVelvet either failed to complete, or recovered an insignificant genome fraction, and so are not reported.

The bacterial community consisted of four species randomly chosen from the well-known MBARC-26 mock community (Singer, 2016): *Terriglobus roseus*, *Salmonella bongori*, *Fervidobacterium pennivorans* and *Sediminispirochaeta smaragdinae*; plus two strains simulated from *S.bongori*, and four strains simulated from *F.pennivorans*. Each simulated strain had between 99.98% and 100.00% of bases aligning to the genome it was simulated from, with an average alignment identity of between 97.76% and 99.44%. Using CAMISIM, we simulated 14.9 Gb of 150 bp paired-end reads, with the abundances as shown in Figure 4A. Table 6 shows the best assemblies from each assembler we tested, with parameters: MEGAHIT (paired, default parameters), MetaCortex (*k* = 63, min coverage = 5), metaSPAdes (default parameters), MetaVelvet (*k* = 95) and Ray Meta (paired, *k* = 31).

**Table 6.** Summary statistics for assemblies of simulated bacterial community

	Genome fraction (%)	Genomes covered > 50%	Contigs	Misassemblies	Mismatch rate	Indel rate
MEGAHIT	65.786	6/10	6602	128	420.37	2.85
MetaCortex	71.276	6/10	5119	94	446.07	5.32
metaSPAdes	54.308	4/10	273	1	110.58	2.13
MetaVelvet	54.027	5/10	6904	8	235.81	4.1
Ray Meta	50.098	4/10	438	4	65.9	0.78

MetaCortex again recovers the highest genome fraction (71%), with 6 out of 10 genomes recovered at at least 50%. This time, the assembly by MEGAHIT contained the most misassemblies, with MetaCortex containing significantly fewer, but still a high misassembly rate. The assemblies by metaSPAdes, MetaVelvet and Ray Meta contained far fewer misassemblies but assembled a much smaller proportion of the genomes present in the sample (27–54%). The assembly by MetaVelvet has the lowest error rates, with no misassemblies, but fails to assemble any sequence for six of the genomes in the community, and for two of the genomes assembles <5%. The assembly by metaSPAdes also has low error rates but recovers significantly less of the genomes than MetaCortex, failing to assemble any of the least two abundant strains (*F.pennivorans*.1 and *F.pennivorans*.2) and <7% of both *F.pennivorans*.3 and *S.bongori*.1.

Both MetaCortex and MEGAHIT had a large number of misassemblies. The majority of misassembled blocks were in contigs aligned to *S.bongori* sim. 2 (the second genome simulated from *S.bongori*), at 65% and 66%, respectively. This genome was the most abundant and had a large read coverage of over 1000×. High coverage is known to cause misassemblies for single species *de novo* genome assembly, with 20×–200× determined the ideal coverage range (Desai, 2013), so we hypothesized that this was the cause of the misassemblies. After subsampling the reads at 10% and reassembling with MetaCortex using the same parameters as before, we found that the number of misassembled blocks belonging to this genome was reduced by 50%. This highlights the difficulty in having a great enough sequencing depth to capture the least abundant genomes. Methods such as digital normalization can be used to address this (Howe, 2014).

### 2.4 Assembly of human gut microbiome sample

The primary focus of MetaCortex is on capturing connected strain-level variation in metagenomic assemblies. However, like other metagenome assemblers, MetaCortex can also be used to obtain metagenome-assembled genomes (MAGs). To demonstrate this, we assembled whole genome shotgun reads sequenced from a human gut microbiome sample. We downloaded 25.1 Gb of reads sequenced using Illumina HiSeq 4000, from the study (Kim, 2021) (SRA run number SRR13060942). Using Trim Galore, we first trimmed adapter sequence and low-quality bases and then assembled the resulting reads using MetaCortex SW (with parameters *k* = 63, min coverage = 2 and SW delta = 0.8). Contigs of length <500 bp were discarded, which left 123720 contigs, with an N50 of 5399 and a maximum length 879838. Using BLAST, we aligned the contigs to the nt database and found 41080 contigs had an alignment covering at least 90% of the contig with an average alignment identity of >95%. Without a ground truth ‘answer’ for this dataset, we were encouraged by this level of similarity to reference sequences, particularly considering there are still many uncharacterized species present in the gut microbiome.

Using Bowtie2 (Langmead and Salzberg, 2012), we mapped the reads against the assembled contigs and binned contigs using MetaBAT2 (Kang, 2019), resulting in 155 genome bins. Since in this case, there was no ground truth to compare our assembly to, we used checkm (Parks, 2015) to assess the bins. This found 23 bins with a completeness level of >50% (min 51.33% and max 91.37%) and maximum contamination of 9.22%. Of these bins, 11 were reported to have high levels of strain heterogeneity (>50%), suggesting that the majority of contamination in these cases is coming from multiple closely related organisms.

### 2.5 Resource usage

We reassembled all datasets and recorded the maximum memory usage and time taken (both real time and total CPU time) for each assembler (Table 7). In all cases, MEGAHIT takes the least time to perform assemblies. This is because MEGAHIT is multithreaded, but this also means that assemblies with MEGAHIT are non-deterministic and therefore not completely reproducible.

**Table 7.** Resource usage statistics for assemblies

Dataset	Assembler	Elapsed time	Total CPU time	Maximum memory usage (GB)
Viral mock A	MEGAHIT	02:01:09	16:09:12	15.67
Viral mock A	MetaCortex	20:05:38	20:05:38	37.57
Viral mock A	metaSPAdes	7-13:33:54	7-13:33:54	142.49
Viral mock A	Ray Meta	3-12:29:55	3-12:29:55	49.80
Viral mock B	MEGAHIT	01:43:23	13:47:04	16.11
Viral mock B	MetaCortex	17:44:49	17:44:49	34.43
Viral mock B	Ray Meta	2-17:40:31	2-17:40:31	29.09
HIV lab mix	MEGAHIT	01:12:37	09:40:56	7.39
HIV lab mix	MetaCortex	05:35:08	05:35:08	31.29
HIV lab mix	metaSPAdes	19:48:48	3-07:15:12	67.92
HIV lab mix	MetaVelvet	04:02:22	04:02:22	37.01
HIV lab mix	Ray Meta	2-00:04:05	2-00:04:05	29.11
Simulated viral	MEGAHIT	01:49:01	14:32:08	11.48
Simulated viral	MetaCortex	1-12:42:56	1-12:42:56	187.54
Simulated viral	metaSPAdes	5-10:30:49	10-21:01:38	113.90
Simulated viral	Ray Meta	5-09:31:57	5-09:31:57	282.82
Simulated bacterial	MEGAHIT	05:51:51	1-22:54:48	11.64
Simulated bacterial	MetaCortex	3-06:13:56	3-06:13:56	150.07
Simulated bacterial	metaSPAdes	4-12:53:31	9-01:47:02	189.53
Simulated bacterial	MetaVelvet	06:32:27	06:32:27	229.37
Simulated bacterial	Ray Meta	4-17:58:07	4-17:58:07	211.61

*Note*: Times are in the format d-hh:mm:ss.

## 3 Algorithm

The main innovation that MetaCortex introduces is two new graph traversal algorithms for metagenomic datasets. The core algorithm, MetaCortex Consensus (MC) is able to produce FASTA, GFA and FASTG outputs. This is the option that should be selected to obtain sequence graphs. The SW algorithm produces only FASTA output but performs much faster than MC and attempts to write a single sequence for each variant that is detected. The user can also choose to only write unitigs (the maximal paths where each inner node has degree 2) as the FASTA output, by specifying the (existing) PerfectPath algorithm.

First, reads are decomposed into k-mers, and a de Bruijn graph is constructed; this graph (which will likely consist of several disconnected subgraphs) can be initially pruned to remove nodes which form short ‘tips’, or which fail to meet a minimum level of coverage. Tips up to a length of 100 are pruned by default, and the default minimum coverage is 2 (these values can be modified with command line options). Following construction of the de Bruijn graph, one of the following metagenomic traversal algorithms is executed. Formal descriptions of the algorithms can be found in the Supplementary Materials.

### 3.1 MetaCortex consensus algorithm

In theory, in a metagenomic read set and given a large enough k-mer size, evolutionarily distinct species whose genomes do not share any k-mers are represented by distinct connected components in the de Bruijn graph. This algorithm seeks to find consensus paths through each connected component of the graph, representing the disparate species in the sample, and then represent the inter-species diversity by looking for local topological variation (e.g. bubbles) along each consensus path, and outputting this in sequence graph format (GFA and FASTQ). Thus, the final output is a sequence graph that represents several related taxa.

**Fig. 5.**
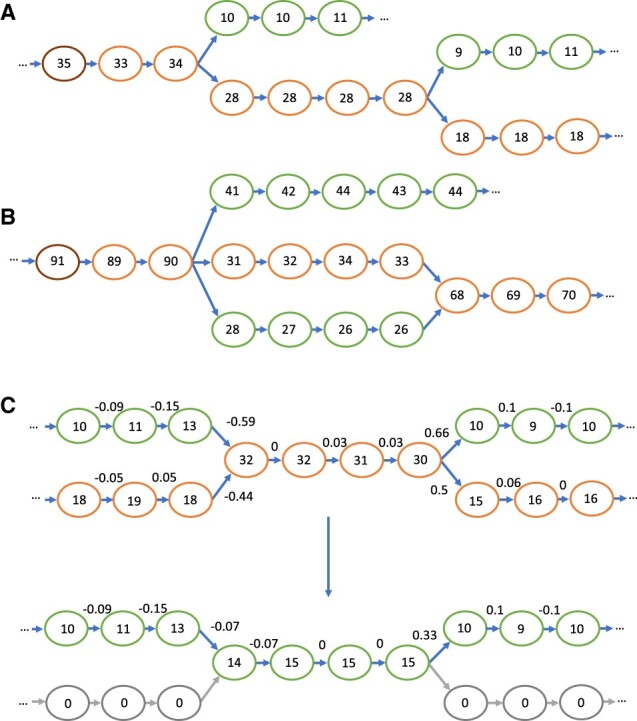
Depictions of de Bruijn graphs, with the coverage for each node represented. (**A**) The path chosen by MetaCortex Consensus, with the highest coverage node on the far left (highlighted in brown), and the chosen path following the lower edges of the graph (highlighted in orange). (**B**) The path chosen when two of the branches form a bubble. Because the two bubble branches, added together, represent a combined higher coverage than the top branch, a route through the bubble is selected for the path. (**C**) The progression of the SW algorithm. The numbers above the edges are the normalized coverage difference/delta. The first graph is before coverage subtraction, with the path chosen again following the lower edges of the graph (highlighted in orange). The second graph is after coverage subtraction. Nodes belonging to exactly one path are removed (now with coverage 0, shown in grey), whilst nodes that are shared between paths remain with reduced coverage. (A color version of this figure appears in the online version of this article)

For each node in the graph, the connected component containing this node is explored to find the highest coverage node and to determine the size of the component. If it is sufficiently large, it is traversed starting at the highest coverage node. At each branching point in the graph, the branch with the highest coverage is favoured if it meets a minimum coverage threshold (Fig. 5A). However, should the paths from two other branches later join together (so that they form a bubble) and have higher coverage collectively, then the highest coverage of these two branches is chosen (Fig. 5B). Traversal continues until a tip is reached, the highest coverage branch at a branching point has already been visited (e.g. in repeat regions), or there are no branches of sufficiently high coverage.

Once a path has been identified, the sequence it represents is written out to a FASTA file. Coverage statistics for the path are included in the header line. If the user has selected to have GFA2/FASTG output, the path is traversed to identify polymorphisms. At each node along the path, any branches that meet a minimum coverage threshold (and are not part of the original path) are explored, depth-first, with the highest coverage node taken at any subsequence branches. If at any point we return to a node on the original path at a position after the original branch, then this new path is written as an alternative path in the GFA file. The path traversal then continues from where the alternative path joins the original path.

Next, each node from the connected component is removed from the graph, and a new connected component is explored. Thus, we obtain one contig and one sequence graph for each sufficiently large connected component.

In many cases however (particularly if *k* is small), disparate species will share k-mers, and it is likely to be the case that the de Bruijn graph consists almost entirely of a single, large, connected component (e.g. Fig. 2A), with several smaller components. To account for this, the ‘-M’ flag can be specified. In this case, during the final node removal step of the algorithm, only nodes in the path are removed, and the remaining nodes in the connected component will be reconsidered. Thus, we obtain multiple contigs for each connected component.

### 3.2 SW algorithm

One of the key difficulties of metagenomic assembly is the presence of multiple distinct strains (or even distinct species) whose genomes share k-mers. This means that in the corresponding de Bruijn graph, some paths may represent portions of the genomes belonging to multiple strains, so it may be desirable to include these in multiple output sequences. The SW algorithm addresses this by not simply removing nodes that have already been traversed, but instead reducing their coverage, so that they may be traversed multiple times.

The algorithm proceeds as follows. First, each node is examined, and for any that meet a minimum coverage requirement, the connected component they are contained in is explored to find the node with locally maximal coverage. (Note that unlike in MC, the whole component may not be explored, in order to speed up the process.) From this node, the highest coverage path is obtained and written to the FASTA file, as in MC, except that at branches, the highest coverage branch is always taken (i.e. bubbles are disregarded).

Next, MetaCortex estimates the number of variants covering each node in the path. First, the lowest coverage node along the path is found, and this is assumed to have one variant covering it. There may be more than one node with the same minimal coverage, in which case, the node closest to the end of the path is chosen. Then, starting from the minimal coverage node, the path is walked in each direction, and at each step, the quantity δ is calculated, where

$$\delta =\frac{c_{\text{current}}-{\;c}_{\text{previous}}}{\text{max}(c_{\text{current}},c_{\text{previous}})}$$

and $c_{\text{current}}$ is the coverage of the node at the current step, and $c_{\text{previous}}$ is the coverage of the node at the previous step. This results in a value between −1 and 1. If this value is less than $-\Delta_{\text{SW}}$ (a value determined by the user with option −W, and set to 0.8 by default) then the number of variants covering this node is assigned the value of the number of variants covering the previous node plus 1; if it is greater than $\Delta_{\text{SW}}$ the number of variants covering this node is assigned whichever is larger of the value of the number of variants at the previous node minus 1, and 1.

After assigning a value to each node estimating the number of variants covering that node in the path, the coverage of nodes is adjusted in the following way. Nodes with 1 variant covering them are reduced to 0 and are essentially removed from the graph. Nodes with more than 1 variant covering them are reduced by an amount which is linearly interpolated from the nearest 1-variant nodes before and after this node in the path (Fig. 5C). This process is repeated until all nodes have been examined.

This algorithm is based on the assumption that, for any two adjacent nodes in the graph that represent k-mers that only appear consecutively in the metagenome (i.e. the first node has outdegree one, and the second node has indegree one), the change in coverage between them will be small compared to their coverage values. On the other hand, for any two adjacent nodes in the graph that represent k-mers that appear consecutively in the metagenome, but at least one of which also appears elsewhere (i.e. at least one of the outdegree of the first node and indegree of the second node is greater than one), the change in coverage between them may be significant compared to the coverage values. The relative change in coverage across the path is what the value δ measures. This assumption, however, is only true for samples that have been sequenced with shotgun metagenome sequencing, and for regions of low sequence complexity (e.g. repeat regions) this may not be the case.

When choosing a value for the parameter $\Delta_{\text{SW}}$, the user should choose smaller values if the dataset is expected to have high levels of strain diversity and the user wishes to capture this in the assembly. For datasets with less strain diversity, or if the user wishes to capture only dominant strains, higher values (closer to 1.0) should be chosen.

## 4 Implementation

MetaCortex uses Cortex’s hash table structure to store k-mer information and to encapsulate the de Bruijn graph structure. For reasons of memory efficiency, the maximum k-mer size must be specified when building MetaCortex. The default maximum value is 31, with 63, 95, 127, 160 or 192 also possible. The size of the hash table is user-defined and should be sufficient to contain the totality of the dataset being assembled (if when loading the reads into the hash table, it becomes full, the user is warned, but execution continues and no new k-mers are added to the hash table). Further details can be found in MetaCortex’s documentation and the cortex_var manual (http://cortexassembler.sourceforge.net/cortex_var_user_manual.pdf, accessed November 2021).

**Fig. 6.**
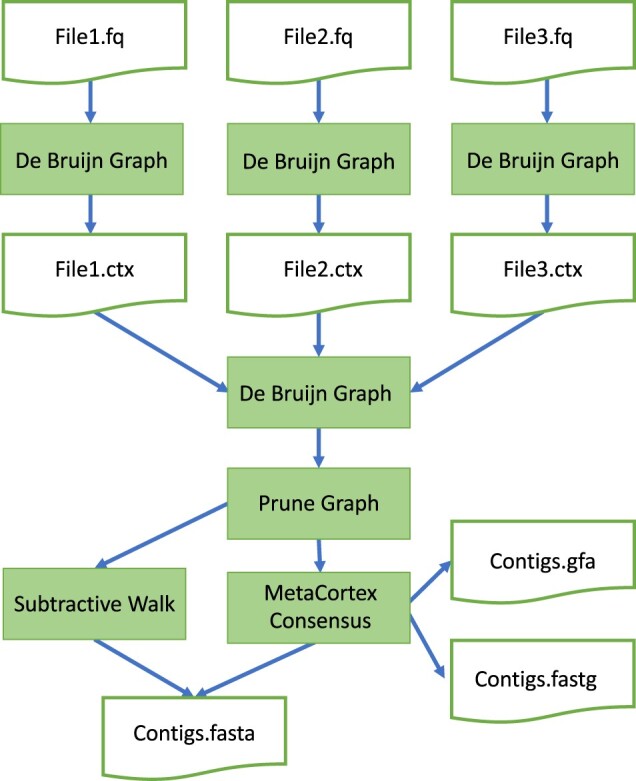
Flow chart depicting typical MetaCortex workflow

After the construction of the hash table from the read files, a binary representation of the de Bruijn graph can be written to disc and this can then be used as the input to later assemblies. This can be used to speed up assembly time for subsequent assemblies of the same dataset, or to parallelize the reading of FASTA or FASTQ files. In the latter case, the individual CTX files can be merged to construct a de Bruijn graph from the whole read set. Figure 6 depicts a typical workflow.

## 5 Discussion

Compared to the challenge of assembling a single isolate species, metagenomic assembly presents significant additional hurdles related to the presence of closely related species and their differing abundance in a sample. These challenges can be particularly obvious in viral communities, where a rapid evolutionary rate can make it especially difficult to distinguish between and within quasispecies. In recent years, several new assembly tools have attempted to tackle the challenges of metagenomic assembly using various heuristic approaches. Most of these have adopted approaches which reduce assembly graphs to sets of contigs, losing the variation captured by the underlying graph structures. While this variation may still be present in an assembly graph, it can be difficult to untangle these graphs for further analysis (as demonstrated in Fig. 2). We address this in MetaCortex, a new assembly tool that preserves strain interconnectedness by outputting sequence graph files as an alternative to contigs. In addition, a new SW algorithm enables MetaCortex to estimate the number of variants in each subgraph and to output representative contigs. This algorithm was able to recover a high proportion of five strains of HIV from a lab mixed community within a metagenomic dataset, and five strains (one real and four simulated) of influenza virus from a simulated viral community, without misassembly. The latter result is particularly encouraging, as several of these strains were at very low abundance in the sample.

Overall, we found that MetaCortex consistently recovers a very high genome fraction when compared to other popular metagenome assemblers. In particular, our simulated datasets show that MetaCortex is especially effective at recovering the genomes of extremely low abundant species. For the assembly of viral communities, MetaCortex had low error rates comparable with the lowest of the other assemblers tested.

## Supplementary Material

btad020_Supplementary_Data

## Data Availability

MetaCortex is open source and available to download from https://github.com/SR-Martin/metacortex, or (for Linux users) via Conda. The version of the source code used for the results presented in this article has DOI 10.5281/zenodo.7273627. Full documentation, including instructions for installation, is available at https://metacortex.readthedocs.io/en/latest/. All assemblies, simulated reads and simulated genomes used in this article have been deposited online on Zenodo and can be found at doi.org/10.5281/zenodo.7298574.
